# The effect of childhood trauma and resilience on psychopathology in adulthood: Does bullying moderate the associations?

**DOI:** 10.1186/s40359-023-01270-8

**Published:** 2023-08-11

**Authors:** Júlia Švecová, Jana Furstova, Natália Kaščáková, Jozef Hašto, Peter Tavel

**Affiliations:** 1 Olomouc University Social Health Institute, Palacky University Olomouc, Univerzitni 22, Olomouc, 77111 Czech Republic; 2 Psychiatric-Psychotherapeutic Outpatient Clinic, Heydukova 27, Bratislava, 81108 Slovakia

**Keywords:** Childhood trauma, Bullying, Resilience, Psychopathology, Moderation

## Abstract

**Background:**

Exposure to traumatic events in childhood, including bullying, can negatively affect physical and mental health in adulthood. The aim of the present study was to determine the prevalence of bullying in different sociodemographic groups of the Slovak Republic and to assess the moderating effect of bullying on the associations between childhood trauma, resilience, and the later occurrence of psychopathology.

**Methods:**

For the analyses, a representative sample of the population of the Slovak Republic was used (N = 1018, mean age 46.24 years, 48.7% of men). Multivariate linear regression models were used to investigate the predictive ability of childhood trauma (The Childhood Trauma Questionnaire, CTQ) and resilience (The Brief Resilience Scale, BRS) to explain psychopathology (The Brief Symptom Inventory, BSI-53). Bullying (The Adverse Childhood Experiences – International Questionnaire, ACE-IQ) was used as a moderator.

**Results:**

In total, 13.5% of respondents have experienced bullying. The most common form of bullying was making fun of someone because of how their body or face looked (46.7%) and excluding someone from activities or ignoring them (36.5%). Higher scores in all types of psychopathology and the Global Severity Index (GSI) were significantly associated with higher scores of emotional and sexual abuse, and some of them with physical neglect. The protective effect of resilience was moderated by bullying in several types of psychopathology, specifically in somatization, obsessive-compulsive, interpersonal sensitivity, depression, psychoticism, and the GSI.

**Conclusion:**

Understanding the links between childhood trauma, bullying, and later psychopathology can help professionals target policies, resources, and interventions to support children and families at risk. Every child should feel accepted and safe at home and school.

**Supplementary Information:**

The online version contains supplementary material available at 10.1186/s40359-023-01270-8.

## Background

“A child should live in peace and in a society that is in the spirit of dignity, tolerance, freedom, equality, and solidarity,” states the Declaration of the Rights of the Child, adopted by the United Nations in 1959 [1]. These values are violated if a child is exposed to traumatic events, including abuse, neglect, or social pain [2]. Five different types of child abuse and neglect are commonly described: physical abuse, emotional abuse, sexual abuse, physical neglect, and emotional neglect [3]. These adverse experiences often emerge from early caregiving relationships; however, they can be accompanied by adverse events from school or other out-of-home environments. Bullying can be considered such a type of traumatic experience [4–7]. It is usually connected to aggressive behaviour like insensitive criticism, ridicule, humiliation, or exclusion from the community. In some cases, bullying even takes the form of physical abuse. The incidence of bullying varies across countries. A 2018 HBSC study [8] found that 12.6% of students from 45 countries were bullied and 3.6% reported being both a bully and a victim of bullying. Northern European countries reported the lowest occurrence of bullying and victimization [9]. Recent research on the experience and behaviour of youth in Slovakia showed that up to a quarter of the children indicated the experience of bullying, with higher prevalence in the age group from 11 to 12 years compared to the group from 15 to 17 years. Girls were victims of bullying more often than boys. Despite the trend of technology, the face-to-face form of bullying still prevails [10]. Research studies on representative or population samples of adults report various results about retrospective experiences of bullying at school age, 6% in the US, 10% in Germany and 18.7% in South Australia [11–13]. Adverse experiences in the family environment have been recognized as risk factors for various forms of psychopathology, including post-traumatic stress disorder (PTSD), depression, anxiety or substance abuse, which can develop already in adolescence [14, 15]. Many research studies confirm that the cumulative effect of adverse childhood experiences (ACE) causes a greater incidence of mental or physical illness symptoms in adulthood [16]. Other researchers have focused on the relationship between the number of adverse experiences in childhood and the likelihood of having physical or psychological difficulties in adulthood. Respondents with four or more ACE were likelier to have somatic and mental health problems [17]. A study by O’Neill et al. [18] placed participants who had experienced domestic violence, physical punishment, emotional abuse and neglect into a “high risk” category for the likelihood of self-harm or becoming a suicide victim. A 2020 meta-analytic study also confirmed the relationship between basic forms of abuse, neglect and suicidal thoughts, plans and attempts [19].

The experience of bullying can aggravate even more the later quality of mental and physical health [20–23]. The most susceptible to PTSD seem to be those bullied who also engaged in the bullying of others (so-called bully victims/aggressive victims). Furthermore, bullying experience can deteriorate the perception of the victim’s body and may distort his/her self-esteem, which may be related to eating disorders in adulthood [24].

Although adverse experiences in childhood may act as a trigger for psychopathology and have often been reported by people who suffer from mental illnesses [25], not everyone who has experienced adverse childhood events will subsequently develop psychopathology. More vulnerable individuals have a higher susceptibility to adverse events [26, 27] and thus are more likely to develop psychopathology [28]. A wide range of psychological constructs with a mediating effect on the relationship between adverse childhood experiences and adult psychopathology have been previously studied [29], and resilience has been recognized as one of the protective factors that can mitigate the risk pathway [30, 31]. Psychological resilience has been defined in several ways. In general, it refers to the process of positive adaptation and recovery from challenging life experiences [32]. Resilience has been receiving great interest among researchers and specialists because of its potential to reduce the negative effect of adverse events and thus prevent the development of stress-related mental disorders [33, 34].

**Fig. 1:**
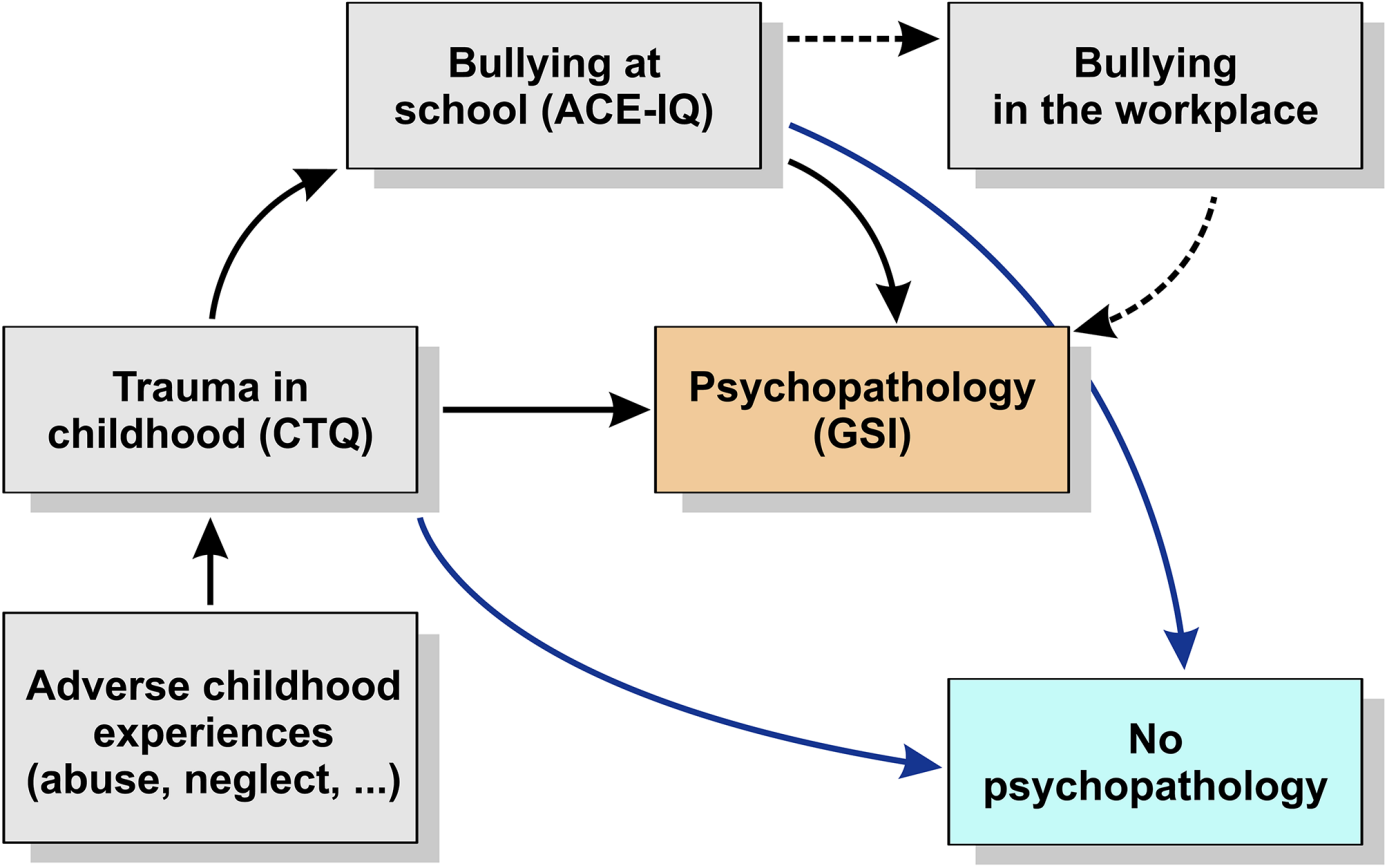
Mutual relations between childhood trauma, bullying and psychopathology

Figure 1 indicates mutual relations between childhood trauma, bullying and psychopathology. We indicated also other lines that show the risk of bullying in the workplace due to the presence of bullying at school and the possible link between bullying in the workplace and the occurrence of psychopathology [35]. We also suggest a possible relationship between the occurrence of psychopathology as a result of traumatic childhood experiences, with or without the experience of bullying. In this model, we also suggest a possibility that includes experiencing trauma or bullying without the subsequent occurrence of psychopathology. The blue line indicates that this can be influenced by the individual´s resilience, which can be supported by personal temperamental characteristics and emotional support from parents, siblings, and peers [36, 37] or corrective experience in other relationships later in adulthood.

Considering the significant associations found in previous research between childhood trauma, resilience, bullying at school age and later psychopathology in adulthood, this study aimed at assessing the prevalence of bullying in different sociodemographic groups and verifying the connections between the given phenomena in a representative adult population of Slovakia. Specifically, the aim was to assess the moderating effect of bullying in the associations between childhood trauma, resilience and the later occurrence of psychopathology. Although this study was designed as a cross-sectional one, from the fact that ACE including bullying were assessed retrospectively and psychopathology was assessed at the present time, we can assume succession in time of these phenomena. We, therefore, hypothesize that experiencing traumatic events together with bullying in childhood can aggravate psychopathology in adulthood.

## Methods

### Research sample

Data collection took place in April 2019 in the form of personal interviews by trained administrators. The sample was selected on the basis of the Statistical Office of the Slovak Republic [38]. The quota characteristics were gender, age, nationality, education, size of place of residence and region of residence, and it is a representative Slovak sample. The research sample consisted of 1,018 respondents, aged 18 to 85 years, average age 46.24 years, 48.7% men. Individual interviews were collected using the CAPI (Computer-Assisted Personal Interviewing) electronic questionnaire [39].

The study was conducted according to the guidelines of the Declaration of Helsinki and approved by the Ethics Scientific Committee of Palacky University Olomouc (No 2019/05), date of approval 5 March 2019.

### Measures

*The Childhood Trauma Questionnaire (CTQ)* is a short questionnaire developed by Bernstein and Fink [40] to measure traumatic experiences in childhood. It can be used with adolescents and adults and includes 5 different types of childhood abuse and neglect: emotional abuse, physical abuse, sexual abuse, emotional neglect and physical neglect. Each of these subscales consists of 5 questions, with every response on a scale from 1 (never) to 5 (very often). The CTQ has 25 subscale questions and 3 questions from the MD scale (Minimization and Denial Scale) which serve to reveal the denial of childhood problems. The scale has been validated on the Slovak population by Petrikova et al. [41]. The Cronbach’s alphas of the CTQ subscales in the present study ranged from 0.64 to 0.94.

*The Adverse Childhood Experiences – International Questionnaire (ACE-IQ)* assesses adverse childhood experiences; it consists of 31 questions and classifies them into 13 areas [42]. For the purpose of this study, only two questions focusing on bullying experiences were used. Specifically, question V1: “How often were you bullied?” with possible answers: Many times – A few times – Once – Never – Refuse to answer; and question V2: “How were you bullied most often?” with possible answers: I was hit, kicked, pushed, shoved around, or locked indoors – I was made fun of because of my race, nationality or colour – I was made fun of because of my religion – I was made fun of with sexual jokes, comments, or gestures – I was left out of activities on purpose or completely ignored – I was made fun of because of how my body or face looked – I was bullied in some other way. The Slovak version of ACE-IQ is currently in the process of validation.

*The Brief Resilience Scale (BRS)* consists of 6 statements. Three of them (1, 3, 5) are positively worded and the other three (2, 4, 6), which are scored by reverse coding, are negatively worded. Respondents indicate on a scale from 1 (strongly disagree) to 5 (strongly agree) how much they agree with the given statement [43]. The scale has been validated on the Slovak population by Furstova et al. [44]. The reliability of the scale on this data was α = 0.86.

*The Brief Symptom Inventory (BSI-53)* is a questionnaire that tracks the occurrence of symptoms of psychopathology over the last 4 weeks [45]. The BSI-53 consists of 53 items, which are rated on a 5-point scale from (0) “not at all” to (4) “extremely”. The questionnaire can be used to monitor the occurrence of 9 psychopathological symptoms/syndromes: Somatization (SOM), Obsessive-Compulsive (O-C), Interpersonal Sensitivity (I-S), Depression (DEP), Anxiety (ANX), Hostility (HOS), Phobic Anxiety (PHOB), Paranoid Ideation (PAR) and Psychoticism (PSY). By calculating the general severity of symptoms (Global Severity Index, GSI), it is possible to assess the current mental state of the respondent [46]. The Slovak version of BSI-53 is currently in the process of validation. The Cronbach’s alphas of individual BSI-53 subscales in this study ranged from 0.83 to 0.90.

### Statistical methods

**Fig. 2:**
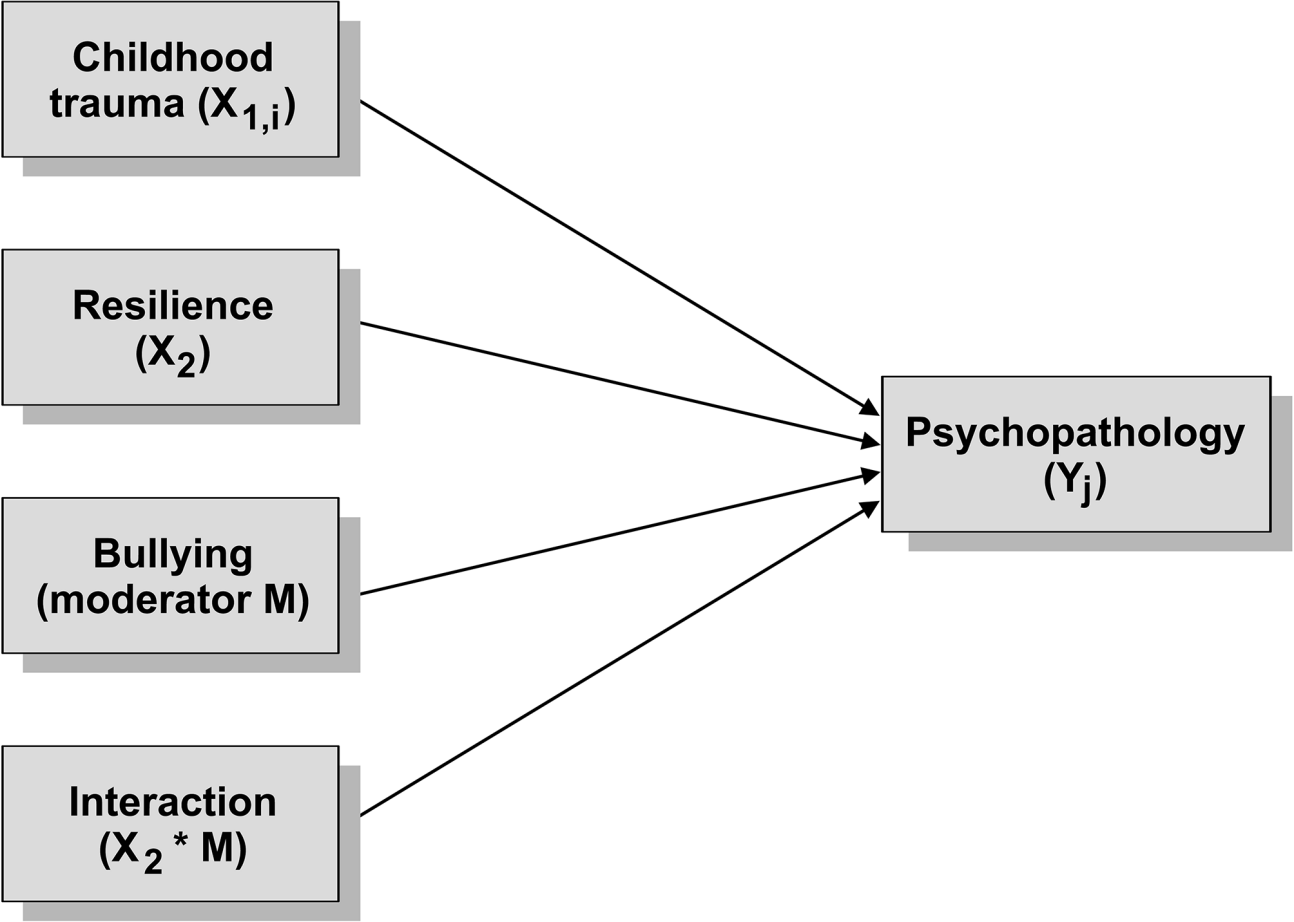
A conceptual framework of the regression models employed in the statistical analyses. Note: *X*_*1,i*_, *X*_*2*_ = the independent variables, where *i = 1,…,5* denotes the individual subscales of the CTQ; *M* = the moderator variable; *X*_2_*M = the interaction of *X*_*2*_ and the moderator variable; *Y*_*j*_=the dependent variable, where *j = 1,…,10* denotes the individual subscales of the BSI-53 and the Global Severity Index (GSI)

All the statistical analyses were performed in the JASP software, version 0.16.2 (JASP Team, University of Amsterdam, The Netherlands). Descriptive statistics and χ^2^ tests in contingency tables were employed as a first step of the analyses. Afterwards, multivariate linear regression models were used to investigate the predictive ability of childhood trauma (CTQ) and resilience (BRS) to explain psychopathology (BSI-53). The moderating effect of bullying was tested by adding an interaction term to the models. The individual subscales of the BSI-53 and the Global Severity Index of psychopathology (GSI) were the dependent variables. All the models were adjusted for the gender and age of the respondents. The conceptual framework of the regression models is presented in Fig. 2. Due to testing multiple models, the significance level was set to α = 0.005. In the Results section, Table 2 contains a brief report of the results. A full result report is presented in Supplementary Tables 1–10.

## Results

Background characteristics of the research sample as well as the occurrence of bullying are presented in Table 1. The prevalence of bullying in the whole sample was 13.5%. The only significant difference in the occurrence of bullying was found in the living arrangement (p = 0.005), as the prevalence of bullying was the lowest in the group of respondents living in a marriage. The majority (60.6%) of those who were bullied reported being bullied several times; a third (32.9%) were bullied once, and 6.6% were bullied often. The most common forms of bullying were making fun of someone because of how his/her body or face looked (46.7%) and leaving someone purposely out of activities or completely ignoring them (36.5%). There were no significant differences found in the frequency or form of bullying between males and females.

**Table 1:** Descriptive characteristics of the research sample

	N	%	Occurrence of bullying (%)	p-value^a^
Total	1018	100	13.5	
**Gender**				0.550
Male	496	48.7	14.1	
Female	522	51.3	12.8	
**Age**				
18–24 y.	110	10.8	13.6	0.651
25–34 y.	187	18.4	13.9	
35–44 y.	199	19.5	13.1	
45–54 y.	166	16.3	15.7	
55–64 y.	168	16.5	16.5	
65 y. and more	188	18.5	14.9	
**Living arrangement**				0.005
Alone	162	15.9	14.8	
With parents	185	18.2	14.6	
With a partner	120	11.8	21.7	
In marriage	551	54.1	10.9	
**Education**				0.023
Primary school	137	13.5	19.7	
Completed apprenticeship	272	26.7	15.8	
Secondary school	382	37.5	11.8	
University or college	227	22.3	9.7	
	**Total**	**Male**	**Female**	
	**N (%)**	**N (%)**	**N (%)**	**p-value** ^**a**^
**Frequency of bullying** ^**b**^				0.168
Often	9 (6.6)	3 (4.3)	6 (9.0)	
Several times	83 (60.6)	42 (60.0)	41 (61.2)	
Once	45 (32.9)	25 (35.7)	20 (29.9)	
**Type of bullying experienced** ^**b**^				
Hit, kicked, pushed, shoved around, or locked indoors	26 (19.0)	18 (25.7)	8 (11.9)	0.040
Made fun of because of race, nationality or color	12 (8.8)	5 (7.1)	7 (10.4)	0.494
Made fun of because of religion	9 (6.6)	7 (10.0)	2 (3.0)	0.098
Made fun of with sexual jokes, comments, or gestures	8 (5.8)	3 (4.3)	5 (7.5)	0.428
Left out of activities on purpose or completely ignored	50 (36.5)	25 (35.7)	25 (37.3)	0.846
Made fun of because of how his/her body or face looked	64 (46.7)	29 (41.4)	35 (52.2)	0.205
Bullied in some other way	36 (26.3)	15 (21.4)	21 (31.3)	0.188

Note. ^a^ P-value corresponds to the χ^2^ test; ^b^ Of those who were bullied.

Table 2 and Supplementary Tables 1–10 show the effect of childhood trauma and resilience on psychopathology, moderated by bullying. Higher scores in all types of psychopathology (BSI-53 subscales) and the Global Severity Index (GSI) were significantly associated with higher scores of Emotional and Sexual abuse. Physical neglect was significantly associated with higher scores in most types of psychopathology and GSI, except for Interpersonal sensitivity, Depression and Paranoid ideation. Physical abuse and Emotional neglect did not have any significant associations with psychopathology. Resilience, as an independent predictor, significantly reduced the scores in all types of psychopathology and GSI. However, the protective effect of resilience was moderated by bullying in several types of psychopathology, specifically in Somatization, Obsessive-compulsive, Interpersonal sensitivity, Depression, Psychoticism and GSI. In these types of psychopathology, the respondents who were bullied had higher scores of psychopathology than those who were not bullied and had the same level of resilience.

**Table 2:** Results of multivariate linear regression models, testing the effect of childhood trauma (CTQ) and resilience (BRS), moderated by bullying, on psychopathology (BSI-53). A separate model was fitted for each subscale of the BSI-53 and the Global Severity Index of psychopathology (GSI). For more detailed results, see Supplementary Tables 1–10

	CTQ subscales							
BSI-53 subscales	EA ^a^	PA ^a^	SA ^a^	EN ^a^	PN ^a^	BRS ^a^	Bullying	BRS*Bullying
Somatization	0.160 ***	0.101 *	0.155 ***	-0.013	0.130 **	-0.167 ***	0.543 **	-0.179 **
Obsessive–compulsive	0.242 ***	0.050	0.114 **	-0.053	0.125 **	-0.191 ***	0.616 ***	-0.173 **
Interpersonal sensitivity	0.364 ***	-0.103 *	0.170 ***	-0.032	0.092 *	-0.205 ***	0.920 ***	-0.250 ***
Depression	0.238 ***	-0.012	0.173 ***	0.001	0.103 *	-0.214 ***	0.799 ***	-0.235 ***
Anxiety	0.257 ***	-0.040	0.211 ***	-0.029	0.150 ***	-0.226 ***	0.545 ***	-0.149 *
Hostility	0.269 ***	-0.003	0.212 ***	-0.032	0.141 ***	-0.145 ***	0.386 *	-0.093
Phobic anxiety	0.123 **	0.098 *	0.281 ***	-0.017	0.152 ***	-0.150 ***	0.330 *	-0.101 *
Paranoid ideation	0.335 ***	-0.055	0.147 ***	0.007	0.050	-0.182 ***	0.594 **	-0.135 *
Psychoticism	0.161 ***	0.067	0.265 ***	-0.009	0.147 ***	-0.153 ***	0.492 **	-0.161 **
Global severity index (GSI)	0.260 ***	0.015	0.209 ***	-0.022	0.137 ***	-0.203 ***	0.561 ***	-0.161 ***

Note: ^a^ Standardized coefficient (computed for continuous predictors only); CTQ = Childhood Trauma Questionnaire; EA = Emotional Abuse; PA = Physical Abuse; SA = Sexual Abuse; EN = Emotional Neglect; PN = Physical Neglect; BRS = Brief Resilience Scale; *p < 0.05; **p < 0.005; ***p < 0.001; All models were adjusted for gender and age.

## Discussion

This study aimed at assessing the prevalence of bullying in different sociodemographic groups and verifying the connections between childhood trauma, resilience, bullying and the later occurrence of psychopathology in a representative adult population of the Slovak Republic. The main findings of the study were: (1) The only significant difference in the occurrence of bullying was found in the living arrangement of respondents; (2) The protective effect of resilience was moderated by bullying in several types of adulthood psychopathology.

### Sociodemographic background and the most common types of bullying in Slovakia

In the present study, the lowest prevalence of bullying was reported in the group of people living in a marriage. This finding may be related to the fact that bullied people tend to trust people less and may find it difficult to get married. However, the results of several previous studies have shown that the connection between bullying and marital status is somewhat ambiguous. The correlation between bullying and satisfaction in romantic relationships was not confirmed in the study by Jantzer et al. [47]. In contrast, a study by Kretchmer et al. [48] found that victims of bullying are less able to handle tasks typical of early adulthood, such as functioning in a romantic relationship, educating, working, managing finances or leadership. The experience of bullying in Slovakia also seems to be connected to the level of education, although without statistical significance. The most bullied in our study were respondents with a completed primary education. It is well known that lower education is associated with socioeconomically disadvantaged groups [49] who also tend to be at higher risk of involvement in bullying, being a bully or a victim [50, 51]. Another disadvantaged group can be adolescents with ADHD. When these students experience bullying, their capability to learn can be compromised [52].

The most common way of bullying in the present study was making fun of someone because of how their body or face looked, which was also confirmed in the Slovak HBSC study [53]. The experience of bullying has a negative impact on the perception of one’s own body and can cause self-image distortion [54]. A 14-year-long longitudinal study [24] showed that most bullied children have feelings of dissatisfaction with their bodies until adulthood, and later they may also develop an eating disorder. The second most common form of bullying in our study was exclusion from participating in collective activities. The prevalence of this form of bullying in Slovakia corresponds to the prevalence of social bullying reported by adolescents in an HBSC study performed in the USA [55]. This type of bullying can have a particularly harmful effect on the victim because social bullying and exclusion from the community has previously been identified as one of the predictors of suicidal behaviour [56]. According to Meltzer et. all [57], who also took into account life factors that reduce the risk of suicidal behaviour, respondents who experienced childhood bullying were more than twice as likely to attempt suicide in adulthood compared to adults who were not bullied.

### Childhood trauma, resilience and psychopathology

In the present study, emotional and sexual abuse predicted higher scores in all types of psychopathology and the Global Severity Index (GSI). A previous study by Rehan et al. [58] showed that even a single experience of emotional or sexual abuse in childhood leads to an increase in psychopathology symptoms in adulthood compared with no experience of abuse. Emotional abuse can be especially detrimental because it may not have immediate visible signs of harm and may remain unrecognized for a considerable time [59]. Emotional abuse and an overly controlling parenting style lead to the development of psychopathology, influenced by low self-esteem and immature defences [60]. A higher frequency of emotional abuse has an impact on the higher occurrence of psychopathology later. On the other hand, the intensity of emotional abuse can negatively influence caregiving representations in terms of negative, critical and dissatisfied statements about their own child [61]. In addition, emotional abuse affects the quality of verbal and social skills, brain development and a person’s hormonal functioning [62]. The impact on changes in specific areas of the brain (amygdala, hippocampus, corpus callosum) is also influenced by the form of neglect or abuse, as well as the age when the adverse experiences took place, i.e. early childhood, puberty or adolescence [63]. The findings above demonstrate that it does matter what type of maltreatment a person is dealing with.

Compared to the above, a clinical study by Noll et al. [64] showed connections between sexual abuse and the later occurrence of psychiatric disorders (such as PTSD, substance abuse, anxiety) and personality disorders, as well as sexual dysfunctions, sexually challenging behaviour and teenage pregnancy. In the language of the brain, the corpus callosum area of the sexually abused girls appears to be susceptible to change, and the genital representational area in the somatosensory cortex becomes thinner [63]. The brain thus attempts to adapt to the hostile surroundings, but in a healthy environment, these functional changes of the brain may manifest as a pathology that needs to be treated [65].

According to our results, physical neglect was another significant predictor of almost all types of psychopathology and GSI. In most cases, failure to provide the basic needs of a child, such as sustenance, medical care or clothing, is related to the poverty of the family. Higher affluence significantly reduces the incidence of child neglect and abuse [66]. Existential stress and financial problems can cause psychological problems, such as depression, alcohol or drug abuse or other mental illnesses, and vice versa, a clinical diagnosis can be the cause of poor economic condition due to the inability to work [67, 68]. Zlotnick et al. [69] observed higher levels of alexithymia, i.e. a dysfunction in identifying, expressing, and cognitively processing emotions, in respondents who experienced emotional and physical neglect than in respondents who were abused. In our study, physical abuse was not associated with higher scores of psychopathology or GSI when used in a multivariate model. In all univariate models, with physical abuse being the only predictor, the association with psychopathology and GSI was significant. However, in the multivariate models where other ACE are present in the model, the effect of physical abuse diminished to a level of statistical insignificance. In our calculations, emotional and sexual abuse prevailed, they were stronger predictors of psychopathology than physical abuse. This is in line with the findings of Iffland et al. [70] who reported that individuals with a history of emotional maltreatment (i.e. emotional abuse or neglect) showed higher rates of all types of psychopathology compared to the respondents who reported a history of physical maltreatment (i.e. physical abuse or neglect). Further, the incidence of social anxiety in adulthood among respondents who experienced physical maltreatment was significantly moderated by the presence of emotional maltreatment [70]. Wright et al. [71] found out that individuals reporting exclusively emotional abuse and neglect have had higher rates on almost all subscales of the BSI. This may be influenced by the fact, that devaluating words has a strong impact on self-worth and self-esteem. In the Slovak Republic, there could be another reason for the diminishing effect of physical abuse on psychopathology: in the adult population, physical abuse in the form of corporal punishment of children by their parents or caregivers is still widely accepted. By comparison, a Finnish study [58] reported that even a single experience of sexual and emotional abuse increased psychopathology, while a single experience of physical abuse did not. However, repeated experiences of abuse affected the occurrence of psychopathology in all three forms, emotional, sexual, and physical [58].

Our results suggest that resilience is a protective factor for the occurrence of all types of psychopathology despite traumatization. For gaining resilience, it is important to have one’s resources (i.e. protective factors) at three levels: personal, socio-cultural, and the wider social environment [72]. Protective factors enhancing resilience can be education, active coping, optimism, interpersonal and emotional competence, social attachment, and support from the family [59, 73]. Developing these in the presence of adversities is thus very important for successful adaptation. The effect of resilience as a protective factor for adolescent mental health and its important role between childhood trauma and the occurrence of later psychopathology symptoms has already been widely recognized in the literature [74–76].

### The moderating effect of bullying

The results of this study show that the effect of resilience was moderated by bullying, i.e. at the same level of resilience, the respondents who were bullied had higher scores in somatization, obsessive-compulsive, interpersonal sensitivity, depression, psychoticism, and the global severity index of psychopathology. If we perceive bullying as a traumatic experience, our results confirm the effect of cumulative trauma. Hodges et al. [77] state that respondents who experience more traumatic events in the same time period later show complexity of symptoms. Our results are in line with Afifi et al. [78], who found that the use of addictive substances by adolescents increases if, in addition to adverse experiences with guardians, they have also experienced bullying by peers. The Glassner and Cho [79] study had similar results. They claimed that the experience of bullying in childhood has an effect on bad moods or blues in adolescence which leads to emotional problems in early adulthood and is associated with a significant increase in substance use in adulthood for both sexes [79]. A study by Bond et al. [80] calls for the implementation of bullying prevention in all schools due to the cumulative effect of adverse experiences in different environments (home, school), which predicts a greater impact on the occurrence of later psychopathology, including suicide attempts.

Many studies have shown a link between bullying in childhood and child maltreatment in the family environment. A large US study on 37,000 school-aged children [81] found a strong relationship between the presence of maltreatment, sexual abuse or neglect in the family environment and the odds that a child/adolescent will be bullied in the school environment. At the same time, “bullies” increasingly report domestic violence, neglect and problems with parental substance abuse [82]. It follows that children who experience domestic violence find themselves in the position of a victim or an aggressor in the process of bullying more often.

Coping with adverse childhood experiences, including bullying, appears to be substantial for future health and well-being. According to previous studies, relationships in the family play a crucial role. If a child’s basic needs are not met in the family, if a child does not feel that he or she belongs there or does not feel safe, and if this frustration is then repeated at school, among peers, or later at work, feelings of loneliness can result in psychopathology [83–85]. When the experience of bullying creates traumatic memories perpetuating the psychopathology in adolescence or adulthood, therapeutic practices processing the trauma (e.g. EMDR, NET TF-CBT or imagery rescripting) can be useful [86–89]. Adolescents with a stronger attachment towards their community, at home and at school, are also less likely to be involved in aggressive behaviour like bullying [51]. On the other hand, if a child has a positive relationship with his or her guardians, good mental abilities and is able to regulate his or her emotions, he or she has a good prerequisite for the development of resilience, despite adverse events [90]. The same holds for adults reporting that they were bullied at school-age: the resilience can be reinforced by having corrective experience in a relationship where they feel safe, accepted and where they get encouragement [91]. As child abuse can lead to intimidating behaviour and deficits in assertiveness in some individuals [92], they can more easily become victims of bullying. It would be appropriate for teachers and educators to develop comprehensive empathy in terms of mentalization [93] and the ability to be a role model in appropriate assertive behaviour [94]. In general, mentalizing is the ability to understand the world of students and at the same time to be in contact with one’s inner self. Bullied individuals need help in developing their assertiveness; they need energetic support from adults and also from the part of their peers who are compassionate and empathetic. Children with a secure relationship are capable of mentalizing as well; therefore, supporting their status in the classroom could contribute to a healthier functioning of the classroom community.

The results of this study highlight the need to promote comprehensive prevention of the long-term harmful effects associated with the dysfunctional relationships in families and socio-pathological behaviour at schools and other children’s facilities. Understanding the links between childhood trauma and later psychopathology could help social work professionals and counsellors target interventions supporting families at risk. Given that majority of mental disorders have their onset by early adulthood, i.e. before the age of 25 [95], the Slovak mental health care system should be targeted towards supporting the young population. Specialists can advocate for policies and resources to help meet the needs of children and families at risk. Further, preventive programs and the activities of counselling and school psychologists play an important role. They can help capture bullying at an early stage, and their actions can be prompt. The occurrence of undesirable phenomena depends on the climate in the individual classrooms and the overall atmosphere in the school; therefore, new approaches in the prevention of bullying and other pathological phenomena are currently focusing on the concept of a “safe school” and anti-bullying class norms [96]. Every child should feel accepted and safe at home and at school. For optimal functioning and effectiveness of preventive efforts in all areas, from primary to tertiary prevention interdepartmental cooperation is important. Professionals working in schools need to have access to methodological guidance, for example, from Educational and Psychological Counselling Centers, as well as the opportunity to meet with colleagues from the Central Office of Labour, Social Affairs and Family, as well as other organizations dedicated to the prevention of socio-pathological phenomena, in order to network and exchange information and ideas. When dealing with adult clients with psychopathological symptoms who seek professional help, it is appropriate to address experiences of bullying within the anamnesis as they may serve as a source for traumatic memories and contribute to the maintenance of psychopathology symptoms [88, 89].

### Limitations of the study

A limitation of this study could be the length and complexity of the test battery. For some respondents, the whole data collection process could be tedious and could lead to fatigue and problems with attention. Further, the data acquisition was conducted through a standardized face-to-face interview in which the respondent’s answers could be affected by the social desirability bias [97]. Another limitation is that the experience of childhood trauma and bullying was self-reported retrospectively, which may cause a response bias [98, 99]. Although retrospective self-reports could be inaccurate by omissions, distorted memories or an unwillingness to report past adversities, some researchers claim that retrospective studies have a legitimate place in research [100]. Further limitation would be relying on an expected validity of two questionnaires used in the study: the ACE-IQ and BSI-53 have not been previously validated in the Slovak environment and are currently in the process of validation. Also, resilience is a complex phenomenon, but we only had limited data for this research from the Brief Resilience Scale (BRS), which allowed us to draw only limited conclusions. On the other hand, the strength of this study would be that the assessment was performed on a representative sample of the Slovak population.

## Conclusion

This study assessed the associations between childhood trauma, resilience, bullying and the later occurrence of psychopathology in a representative adult population of the Slovak Republic. The most important finding of the study was that the protective effect of resilience was moderated by bullying in several types of adulthood psychopathology. Understanding the links between childhood trauma, bullying and later psychopathology could help professionals target policies, resources and interventions towards those at risk. Further, school psychologists can help identify and address bullying at schools and promote comprehensive prevention programs to mitigate aggressive behaviour.

### Electronic supplementary material

Below is the link to the electronic supplementary material.

Supplementary Material 1: Tables 1–10 show more detailed results of multivariate linear regression models, testing the effect of childhood trauma (CTQ) and resilience (BRS), moderated by bullying, on psychopathology (BSI-53). For each subscale of the BSI-53 and the Global Severity Index of psychopathology (GSI), a separate model was fitted and its results are presented in a separate table.

## Data Availability

The datasets generated and analyzed during the current study are not publicly available but are available from the corresponding author upon reasonable request.

## List of abbreviations

ACE: Adverse Childhood Experiences
ACE- IQ: Adverse Childhood Experiences – International Questionnaire
ADHD: Attention Deficit Hyperactivity Disorder
ANX: Anxiety
BRS: Brief Resilience Scale
BSI-53: Brief Symptom Inventory
CAPI: Computer-Assisted Personal Interviewing
CTQ: Childhood Trauma Questionnaire
DEP: Depression
GSI: Global Severity Index
HBSC: Health Behaviour in school-aged Children
HOS: Hostility
I-S: Interpersonal Sensitivity
MD-scale: Minimization and Denial Scale
O-C: Obsessive-Compulsive
PAR: Paranoid Ideation
PHOB: Phobic Anxiety
PSY: Psychoticism
PTSD: Post-traumatic stress disorder
SOM: Somatization
USA: United States of America
US: United Sates
WHO: World Health Office
