# Supplementary material for: The effect of childhood trauma and resilience on psychopathology in adulthood: Does bullying moderate the associations?
Source: BMC Psychol. 2023 Aug 11;11:230. doi: 10.1186/s40359-023-01270-8 (PMC10422767; doi:10.1186/s40359-023-01270-8)
Supplement: Supplementary file 1 — Supplementary Material 1: Tables 1–10 show more detailed results of multivariate linear regression models, testing the effect of childhood trauma (CTQ) and resilience (BRS), moderated by bullying, on psychopathology (BSI-53). For each subscale of the BSI-53 and the Global Severity Index of psychopathology (GSI), a separate model was fitted and its results are presented in a separate table. [file 40359_2023_1270_MOESM1_ESM.docx]

**Supplementary Tables 1–10**

Supplementary Table 1: Somatization (R^2^=0.388)

| BSI-53 Somatization | **Beta coef. ^a^** | **Standard Error** |  | **95% CI** | |
| --- | --- | --- | --- | --- | --- |
| **Predictors** |  |  | **p-value** | **Lower** | **Upper** |
| CTQ EA | 0.160 | 0.009 | < .001 | 0.017 | 0.052 |
| CTQ PA | 0.101 | 0.011 | 0.023 | 0.004 | 0.048 |
| CTQ SA | 0.155 | 0.011 | < .001 | 0.023 | 0.065 |
| CTQ EN | -0.013 | 0.005 | 0.719 | -0.011 | 0.008 |
| CTQ PN | 0.130 | 0.008 | 0.001 | 0.011 | 0.043 |
| BRS | -0.167 | 0.022 | < .001 | -0.175 | -0.088 |
| Bullying | 0.543 | 0.170 | 0.001 | 0.209 | 0.878 |
| BRS*Bullying | -0.179 | 0.057 | 0.002 | -0.292 | -0.066 |
| Age | 0.273 | 0.000 | < .001 | 0.008 | 0.012 |
| Gender | 0.061 | 0.031 | 0.051 | -21.330 | 0.122 |
| Note: ^a^ In case of continuous predictors coefficients are standardized; CTQ = Childhood trauma questionnaire; EA = Emotional abuse; PA = Physical abuse; SA = Sexual abuse; EN = Emotional neglect; PN = Physical neglect; BRS = Brief resilience scale; Adjusted for gender and age. | | | | | |

Supplementary Table 2: Obsessive–compulsive (R^2^=0.339)

| BSI-53 Obsessive–compulsive | **Beta coef. ^a^** | **Standard Error** |  | **95% CI** | |
| --- | --- | --- | --- | --- | --- |
| **Predictors** |  |  | **p-value** | **Lower** | **Upper** |
| CTQ EA | 0.242 | 0.009 | < .001 | 0.033 | 0.069 |
| CTQ PA | 0.050 | 0.012 | 0.280 | -0.010 | 0.035 |
| CTQ SA | 0.114 | 0.011 | 0.003 | 0.011 | 0.053 |
| CTQ EN | -0.053 | 0.005 | 0.152 | -0.017 | 0.003 |
| CTQ PN | 0.125 | 0.008 | 0.002 | 0.009 | 0.042 |
| BRS | -0.191 | 0.023 | < .001 | -0.191 | -0.102 |
| Bullying | 0.616 | 0.173 | < .001 | 0.276 | 0.956 |
| BRS*Bullying | -0.173 | 0.058 | 0.003 | -0.288 | -0.059 |
| Age | 0.187 | 0.000 | < .001 | 0.005 | 0.009 |
| Gender | 0.020 | 0.032 | 0.523 | -0.042 | 0.082 |
| Note: ^a^ In case of continuous predictors coefficients are standardized; CTQ = Childhood trauma questionnaire; EA = Emotional abuse; PA = Physical abuse; SA = Sexual abuse; EN = Emotional neglect; PN = Physical neglect; BRS = Brief resilience scale; Adjusted for gender and age. | | | | | |

Supplementary Table 3: Interpersonal sensitivity (R^2^=0.368)

| BSI-53 Interpersonal sensitivity | **Beta coef. ^a^** | **Standard Error** |  | **95% CI** | |
| --- | --- | --- | --- | --- | --- |
| **Predictors** |  |  | **p-value** | **Lower** | **Upper** |
| CTQ EA | 0.364 | 0.010 | < .001 | 0.066 | 0.104 |
| CTQ PA | -0.103 | 0.013 | 0.023 | -0.053 | -0.004 |
| CTQ SA | 0.170 | 0.012 | < .001 | 0.029 | 0.075 |
| CTQ EN | -0.032 | 0.005 | 0.380 | -0.015 | 0.006 |
| CTQ PN | 0.092 | 0.009 | 0.023 | 0.003 | 0.038 |
| BRS | -0.205 | 0.024 | < .001 | -0.221 | -0.126 |
| Bullying | 0.920 | 0.187 | < .001 | 0.554 | 1.286 |
| BRS*Bullying | -0.250 | 0.063 | < .001 | -0.373 | -0.126 |
| Age | 0.084 | 0.001 | 0.001 | 0.001 | 0.005 |
| Gender | 0.083 | 0.034 | 0.015 | 0.016 | 0.150 |
| Note: ^a^ In case of continuous predictors coefficients are standardized; CTQ = Childhood trauma questionnaire; EA = Emotional abuse; PA = Physical abuse; SA = Sexual abuse; EN = Emotional neglect; PN = Physical neglect; BRS = Brief resilience scale; Adjusted for gender and age. | | | | | |

Supplementary Table 4: Depression (R^2^=0.352)

| BSI-53 Depression | **Beta coef. ^a^** | **Standard Error** |  | **95% CI** | |
| --- | --- | --- | --- | --- | --- |
| **Predictors** |  |  | **p-value** | **Lower** | **Upper** |
| CTQ EA | 0.238 | 0.009 | < .001 | 0.033 | 0.069 |
| CTQ PA | -0.012 | 0.012 | 0.787 | -0.026 | 0.020 |
| CTQ SA | 0.173 | 0.011 | < .001 | 0.028 | 0.071 |
| CTQ EN | 0.001 | 0.005 | 0.974 | -0.010 | 0.010 |
| CTQ PN | 0.103 | 0.008 | 0.012 | 0.005 | 0.038 |
| BRS | -0.214 | 0.023 | < .001 | -0.213 | -0.123 |
| Bullying | 0.799 | 0.175 | < .001 | 0.456 | 1.143 |
| BRS*Bullying | -0.235 | 0.059 | < .001 | -0.351 | -0.119 |
| Age | 0.151 | 0.000 | < .001 | 0.004 | 0.008 |
| Gender | 0.014 | 0.032 | 0.659 | -0.049 | 0.077 |
| Note: ^a^ In case of continuous predictors coefficients are standardized; CTQ = Childhood trauma questionnaire; EA = Emotional abuse; PA = Physical abuse; SA = Sexual abuse; EN = Emotional neglect; PN = Physical neglect; BRS = Brief resilience scale; Adjusted for gender and age. | | | | | |

Supplementary Table 5: Anxiety (R^2^=0.376)

| BSI-53 Anxiety | **Beta coef. ^a^** | **Standard Error** |  | **95% CI** | |
| --- | --- | --- | --- | --- | --- |
| **Predictors** |  |  | **p-value** | **Lower** | **Upper** |
| CTQ EA | 0.257 | 0.008 | < .001 | 0.035 | 0.068 |
| CTQ PA | -0.04 | 0.011 | 0.375 | -0.031 | 0.012 |
| CTQ SA | 0.211 | 0.01 | < .001 | 0.036 | 0.075 |
| CTQ EN | -0.029 | 0.005 | 0.427 | -0.013 | 0.005 |
| CTQ PN | 0.15 | 0.008 | < .001 | 0.014 | 0.044 |
| BRS | -0.226 | 0.021 | < .001 | -0.206 | -0.124 |
| Bullying | 0.545 | 0.16 | < .001 | 0.232 | 0.859 |
| BRS*Bullying | -0.149 | 0.054 | 0.006 | -0.254 | -0.043 |
| Age | 0.109 | 0.000 | < .001 | 0.002 | 0.005 |
| Gender | 0.020 | 0.029 | 0.486 | -0.037 | 0.078 |
| Note: ^a^ In case of continuous predictors coefficients are standardized; CTQ = Childhood trauma questionnaire; EA = Emotional abuse; PA = Physical abuse; SA = Sexual abuse; EN = Emotional neglect; PN = Physical neglect; BRS = Brief resilience scale; Adjusted for gender and age. | | | | | |

Supplementary Table 6: Hostility (R^2^=0.334)

| BSI-53 Hostility | **Beta coef. ^a^** | **Standard Error** |  | **95% CI** | |
| --- | --- | --- | --- | --- | --- |
| **Predictors** |  |  | **p-value** | **Lower** | **Upper** |
| CTQ EA | 0.269 | 0.008 | < .001 | 0.036 | 0.069 |
| CTQ PA | -0.003 | 0.011 | 0.945 | -0.022 | 0.020 |
| CTQ SA | 0.212 | 0.010 | < .001 | 0.035 | 0.074 |
| CTQ EN | -0.032 | 0.005 | 0.382 | -0.013 | 0.005 |
| CTQ PN | 0.141 | 0.008 | < .001 | 0.011 | 0.042 |
| BRS | -0.145 | 0.021 | < .001 | -0.144 | -0.062 |
| Bullying | 0.386 | 0.160 | 0.016 | 0.071 | 0.701 |
| BRS*Bullying | -0.093 | 0.054 | 0.084 | -0.200 | 0.013 |
| Age | 0.034 | 0.000 | 0.207 | -66.770 | 0.003 |
| Gender | -0.047 | 0.029 | 0.106 | -0.105 | 0.010 |
| Note: ^a^ In case of continuous predictors coefficients are standardized; CTQ = Childhood trauma questionnaire; EA = Emotional abuse; PA = Physical abuse; SA = Sexual abuse; EN = Emotional neglect; PN = Physical neglect; BRS = Brief resilience scale; Adjusted for gender and age. | | | | | |

Supplementary Table 7: Phobic anxiety (R^2^=0.372)

| BSI-53 Phobic anxiety | **Beta coef. ^a^** | **Standard Error** |  | **95% CI** | |
| --- | --- | --- | --- | --- | --- |
| **Predictors** |  |  | **p-value** | **Lower** | **Upper** |
| CTQ EA | 0.123 | 0.008 | 0.004 | 0.008 | 0.039 |
| CTQ PA | 0.098 | 0.010 | 0.030 | 0.002 | 0.042 |
| CTQ SA | 0.281 | 0.009 | < .001 | 0.051 | 0.088 |
| CTQ EN | -0.017 | 0.004 | 0.631 | -0.011 | 0.006 |
| CTQ PN | 0.152 | 0.007 | < .001 | 0.013 | 0.041 |
| BRS | -0.150 | 0.020 | < .001 | -0.141 | -0.064 |
| Bullying | 0.330 | 0.150 | 0.028 | 0.036 | 0.625 |
| BRS*Bullying | -0.101 | 0.051 | 0.045 | -0.201 | -0.002 |
| Age | 0.094 | 0.000 | < .001 | 0.001 | 0.005 |
| Gender | 0.030 | 0.027 | 0.275 | -0.024 | 0.084 |
| Note: ^a^ In case of continuous predictors coefficients are standardized; CTQ = Childhood trauma questionnaire; EA = Emotional abuse; PA = Physical abuse; SA = Sexual abuse; EN = Emotional neglect; PN = Physical neglect; BRS = Brief resilience scale; Adjusted for gender and age. | | | | | |

Supplementary Table 8: Paranoid ideation (R^2^=0.299)

| BSI-53 Paranoid ideation | **Beta coef. ^a^** | **Standard Error** |  | **95% CI** | |
| --- | --- | --- | --- | --- | --- |
| **Predictors** |  |  | **p-value** | **Lower** | **Upper** |
| CTQ EA | 0.335 | 0.010 | < .001 | 0.058 | 0.098 |
| CTQ PA | -0.055 | 0.013 | 0.255 | -0.041 | 0.011 |
| CTQ SA | 0.147 | 0.012 | < .001 | 0.021 | 0.069 |
| CTQ EN | 0.007 | 0.006 | 0.855 | -0.010 | 0.012 |
| CTQ PN | 0.050 | 0.009 | 0.238 | -0.007 | 0.030 |
| BRS | -0.182 | 0.026 | < .001 | -0.205 | -0.104 |
| Bullying | 0.594 | 0.197 | 0.003 | 0.208 | 0.979 |
| BRS*Bullying | -0.135 | 0.066 | 0.042 | -0.265 | -0.005 |
| Age | 0.035 | 0.001 | 0.209 | -81.550 | 0.004 |
| Gender | 0.015 | 0.036 | 0.672 | -0.055 | 0.086 |
| Note: ^a^ In case of continuous predictors coefficients are standardized; CTQ = Childhood trauma questionnaire; EA = Emotional abuse; PA = Physical abuse; SA = Sexual abuse; EN = Emotional neglect; PN = Physical neglect; BRS = Brief resilience scale; Adjusted for gender and age. | | | | | |

Supplementary Table 9: Psychoticism (R^2^=0.365)

| BSI-53 Psychoticism | **Beta coef. ^a^** | **Standard Error** |  | **95% CI** | |
| --- | --- | --- | --- | --- | --- |
| **Predictors** |  |  | **p-value** | **Lower** | **Upper** |
| CTQ EA | 0.161 | 0.008 | < .001 | 0.015 | 0.046 |
| CTQ PA | 0.067 | 0.010 | 0.139 | -0.005 | 0.035 |
| CTQ SA | 0.265 | 0.009 | < .001 | 0.047 | 0.084 |
| CTQ EN | -0.009 | 0.004 | 0.801 | -0.010 | 0.007 |
| CTQ PN | 0.147 | 0.007 | < .001 | 0.012 | 0.041 |
| BRS | -0.153 | 0.020 | < .001 | -0.142 | -0.065 |
| Bullying | 0.492 | 0.150 | 0.001 | 0.197 | 0.787 |
| BRS*Bullying | -0.161 | 0.051 | 0.002 | -0.261 | -0.062 |
| Age | 0.074 | 0.000 | 0.005 | 0.000 | 0.004 |
| Gender | 0.022 | 0.027 | 0.421 | -0.032 | 0.076 |
| Note: ^a^ In case of continuous predictors coefficients are standardized; CTQ = Childhood trauma questionnaire; EA = Emotional abuse; PA = Physical abuse; SA = Sexual abuse; EN = Emotional neglect; PN = Physical neglect; BRS = Brief resilience scale; Adjusted for gender and age. | | | | | |

Supplementary Table 10: Global severity index (GSI) (R^2^=0.419)

| BSI-53 Global severity index (GSI) | **Beta coef. ^a^** | **Standard Error** |  | **95% CI** | |
| --- | --- | --- | --- | --- | --- |
| **Predictors** |  |  | **p-value** | **Lower** | **Upper** |
| CTQ EA | 0.260 | 0.008 | < .001 | 0.034 | 0.064 |
| CTQ PA | 0.015 | 0.010 | 0.730 | -0.016 | 0.022 |
| CTQ SA | 0.209 | 0.009 | < .001 | 0.034 | 0.069 |
| CTQ EN | -0.022 | 0.004 | 0.523 | -0.011 | 0.005 |
| CTQ PN | 0.137 | 0.007 | < .001 | 0.011 | 0.038 |
| BRS | -0.203 | 0.019 | < .001 | -0.175 | -0.101 |
| Bullying | 0.561 | 0.144 | < .001 | 0.279 | 0.844 |
| BRS*Bullying | -0.161 | 0.049 | < .001 | -0.256 | -0.065 |
| Age | 0.145 | 0.000 | < .001 | 0.003 | 0.006 |
| Gender | 0.026 | 0.026 | 0.325 | -0.026 | 0.078 |
| Note: ^a^ In case of continuous predictors coefficients are standardized; CTQ = Childhood trauma questionnaire; EA = Emotional abuse; PA = Physical abuse; SA = Sexual abuse; EN = Emotional neglect; PN = Physical neglect; BRS = Brief resilience scale; Adjusted for gender and age. | | | | | |
